# Intravenous and Subcutaneous Toxicity and Absorption Kinetics in Mice and Dogs of the Antileishmanial Triterpene Saponin PX-6518

**DOI:** 10.3390/molecules18044803

**Published:** 2013-04-22

**Authors:** Louis Maes

**Keywords:** PX-6518, maesabalide, triterpene saponins, systemic toxicity, plasma kinetics, mouse, dog

## Abstract

The intravenous (IV) and subcutaneous (SC) toxicity and absorption kinetics of the antileishmanial triterpene saponin PX-6518 and its active constituents maesabalide-III and -IV were studied in mice and dogs. A high-dose wash-out study of PX-6518 at 20 mg/kg SC for 5 days and a single low-dose wash-out study at 1, 2.5 or 5 mg/kg SC and IV with follow-up until day 35 after treatment were performed in mice. Beagle dogs received three escalating doses of maesabalide-III and -IV at weekly intervals (0.01, 0.1 and 0.5 mg/kg IV and maesabalide-III was also dosed SC at 0.1, 0.2 and 0.4 mg/kg). Endpoint measurements included clinical, hematological and serum biochemical parameters. Pathology and toxicokinetic studies were performed on the dogs. Whereas the neutrophils and aspartate aminotransferase and alanine aminotransferase levels were increased in the high-dose wash-out mouse study, these parameters did not change in the low-dose wash-out study. The dogs were far more susceptible than mice to liver toxicity (hepatocellular necrosis and elevated liver enzymes) and developed a painful inflammatory reaction at the SC injection site. Toxicokinetic analysis revealed a non dose-linear systemic availability with plasma concentrations above the antileishmanial IC_50_ after only a single dose at 0.01 mg/kg IV or 0.1 mg/kg SC. Related to the long half-life (T_1/2_ 71–91 h after SC dosing), repeated dosing at weekly intervals may result in drug accumulation and enhanced toxicity. It was decided not to pursue further drug development for PX-6518 because of the hepatotoxic risk.

## 1. Introduction

Saponins occur in a large number of plant species and are believed to have an important regulatory function in plant metabolism and plant microbial disease resistance [1]. Based on the nature of their aglycon skeleton, saponins can be classified into steroidal and triterpenoid saponins [2] to which very different biological and pharmacological activities have been attributed such as hypocholesterolemic, anticoagulant, anticarcinogenic, hepatoprotective, hypoglycaemic, neuroprotective, immunomodulatory, anti-inflammatory and anti-oxidant properties [3,4,5]. On the other hand, membranolytic toxicity, adverse effects on growth and performance, hypocholesterolemic effects, hypertension, and others have also been reported [6,7]. Of practical medical use and probably the best known are the ginsenosides isolated from dried roots and leaves of *Panax ginseng*, a plant widely used in Traditional Chinese Medicine [8] and currently acquiring growing interest in Western medicine [9]. Other saponins with established use in man are licorice root (*Liquiritiae radix*) for peptic ulcers [10], aescin (*Aesculus hippocastanum*) for vascular disease [11], asiaticoside (*Centella asiatica*) as a wound healing agent [12] and quillaria (*Quillaja saponaria*) as a vaccine adjuvant and immunostimulant [13].

Among the wide range of antimicrobial properties that have been reported, including antifungal, antiviral, antibacterial, antiprotozoal, piscicidal, molluscicidal and insect-larvicidal action [4], the present study specifically focused on the antileishmanial oleane triterpene saponin PX-6518 [14], which is a crude methanolic extract of the leaves of a Vietnamese plant *Maesa balansae* (fam. *Myrsinacceae*), consisting of six maesabalides for which potent and selective action against different *Leishmania* species was demonstrated [15]. Structural analogues were also shown to have comparable antileishmanial activity [16,17]. *In vitro* studies with PX-6518 indicated an IC_50_ of 40 ng/mL against intracellular *L. infantum* amastigotes [15], while *in vivo* experiments with *L. donovani* in hamsters demonstrated that a single subcutaneous (SC) dose at 0.2 mg/kg BW was fully effective [18]. PX-6518 also showed prophylactic and curative efficacy against cutaneous leishmaniasis after repeated SC dosing at 1 mg/kg BW in Balb/c mice [19]. In view of the promising antileishmanial potential of PX-6518 and the current need for new antileishmanial drugs, initial steps were taken to explore the “drug candidate” potential of this molecule with particular focus on *in vivo* toxicity and absorption kinetics in (non-infected) mice and dogs.

Saponins have already been shown to possess weak to strong haemolytic activity dependent on the structure [20]. Despite their widespread occurrence in foods, systemic toxicity is low due to their very poor oral bioavailability [21,22]. In view of the fact that PX-6518 needs to be administered parenterally to exert its antileishmanial action [15], repeated-dose toxicity studies supplemented with toxicokinetic analysis were performed in rodent (CD-1 Swiss mouse) and non-rodent (beagle dog) species after both intravenous (IV) and SC administration in an attempt to assess the overall tolerance and to identify the main systemic toxic effects and target organs for toxicity.

## 2. Results and Discussion

In literature, there are a very vast number of reports that deal with saponins and their chemical, pharmacological and toxicological properties. The large chemical diversity could possibly explain the range of different, mostly unrelated properties; however, much caution is required here in view of the known (cyto)toxicity of this class of chemicals [23]. Furthermore, most publications deal with *in vitro* results, which in most instances were not checked for selectivity of action by inclusion of a parallel cytotoxicity evaluation [24]. For example, most of the described antimicrobial activities may possibly be linked to the strong disruptive action of saponins on cell membrane integrity, causing non-specific cell lysis and death [25].

The finding that PX-6518 showed potent and selective *in vitro* activity against intracellular amastigotes of different *Leishmania* species and could subsequently be confirmed in hamster and mouse models of leishmaniasis [15,19] prompted us to design further in-depth investigations as part of a drug development program. Related to the overall poor oral bioavailability of saponins, it was not at all unexpected that PX-6518 was only active after parenteral administration [15], hence specific focus was given to *in vivo* toxicity after IV and SC administration in a rodent (mouse) and non-rodent (beagle dog) laboratory animal species, with toxicokinetic back-up in the dog studies (Table 1). To the author’s knowledge, comparable *in vivo* toxicity information on other saponins is only scarcely available in the public domain.

**Table 1:** Overview of *in vivo* toxicology studies.

Study	Type	Animalsn.	Treatment				Endpoints
			route	freq	dose (mg/kg)		
PX-6518 mixture (in mice)							
*T1*	high-dose wash-out	42	SC	5× (daily)		20	clinic, haematology, serum biochemistry
*T2*	low-dose wash-out	240	SC	1×		0, 1, 2.5, 5	
*T3*	low-dose wash-out	240	IV	1×		0, 1, 2.5, 5	
Maesabalide-III (in dogs)							
*T4*	dose-escalation	4	IV	3× (d0, 7, 14)		0.01, 0.1, 0.5^#^	clinic, haematology, serum biochemistry, urinalysis, gross- and histopathology
*T5*	dose-escalation	4	SC	3× (d0, 14, 28)		0.1, 0.2, 0.4	Idem *T4* + toxicokinetics
Maesabalide-IV (in dogs)							
*T6*	dose-escalation	4	IV	3× (d0, 7, 14)		0.01, 0.1, 0.5	Idem T5

IV = intravenous, SC = subcutaneous; # two animals needed to be killed in extremis 7 days after treatment at 0.5 mg/kg due to toxicity.

### 2.1. Toxicity in Mice

**Figure 1:**
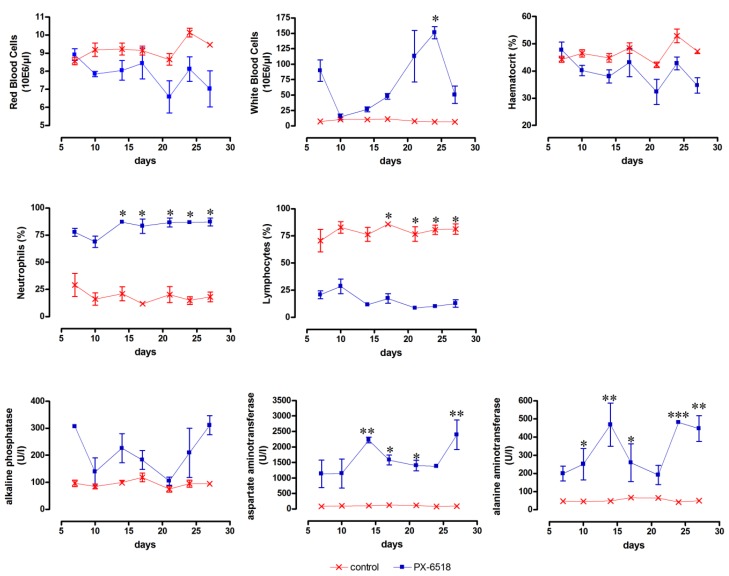
Monitoring of target toxicity parameters (red blood cells, white blood cells, haematocrit, neutrophils, lymphocytes, alkaline phosphatase, aspartate aminotransferase and alanine aminotransferase) in mice after SC dosing at 20 mg/kg for 5 consecutive days. (n = 3 for each time point) * *p* < 0.05, ** *p* < 0.01 and *** *p* < 0.001 (two-way ANOVA with Bonferroni *post-hoc*).

From a prior small-scale pilot toxicity study (data not shown) with a crude extract of PX-6518, it was found that increase of the liver enzymes ALT and AST and a decrease in THR and RBC and increase of WBC (particularly neutrophils) were the target toxicity parameters. The high-dose wash-out study (Study T1*)* indeed confirmed that the target toxicity parameters leucocytosis, granulocytosis, lymphopenia and the increase of the liver enzymes ALP, AST and ALT did not return to normal pre-dose values within 4 weeks after SC dosing (Figure 1), but no deaths occurred and all animals completed the study. The absence of reversibility of the listed toxicity parameters during the 4-week drug wash-out period after a 5-day loading session at 20 mg/kg SC may have resulted from a substantial accumulation of the drug in the body and hence prolonged systemic exposure and toxicity. Indirect indications for the accumulation potential were already obtained from an efficacy study in mice infected with *L. donovani* where a 5-day residual activity period was observed after a single 2.5 mg/kg SC dose [15].

**Figure 2:**
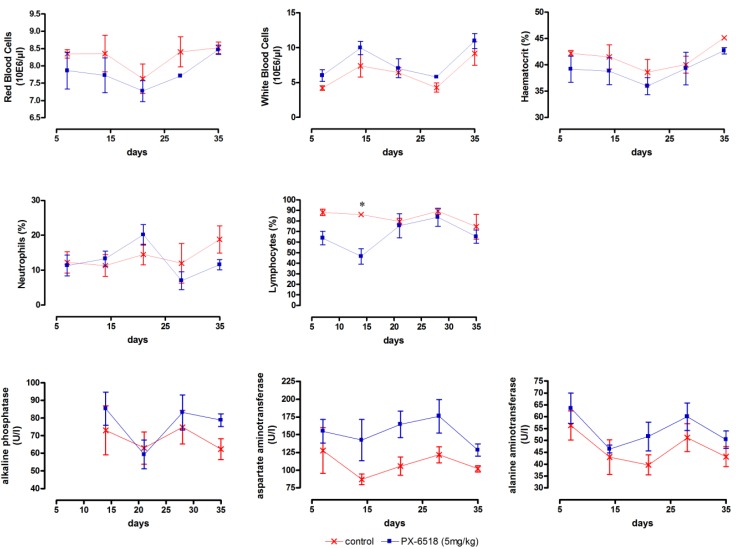
Monitoring of target toxicity parameters (red blood cells, white blood cells, haematocrit, neutrophils, lymphocytes, alkaline phosphatase, aspartate aminotransferase and alanine aminotransferase) in mice after a single SC dose at 5 mg/kg (results for 1 and 2.5 mg/kg did not differ from vehicle-treated control and are not shown). (n = 3 for each time point) * *p* < 0.05, ** *p* < 0.01 and *** *p* < 0.001 (two-way ANOVA with Bonferroni *post-hoc*)*.*

A single-dose study at a much lower dose was the next logical step with particular attention for the liver enzymes. The single-dose schemes in the low-dose wash-out studies were also better in line with the putative future clinical positioning of the drug. No toxicity-related deaths or drug-related clinical side effects occurred in any dosing group and no abnormalities at all developed in the 1 mg/kg and 2.5 mg/kg dosing groups. After SC dosing at 5 mg/kg (Study T2), the liver enzymes ALP, AST and ALT became marginally elevated, but this elevation never reached significance (Figure 2). At 5 mg/kg, a significantly decreased level of lymphocytes was noted only on day 14. The picture after IV dosing (study T3) was principally the same as for SC dosing and results are therefore not shown. Both low-dose studies indicate that a single dose up to 2.5 mg/kg is well tolerated, irrespective the route of administration. Subcutaneous injection caused a transient inflammatory reaction at the injection site and the observed toxic changes of the liver enzyme levels were fully reversible within 2 weeks, indicating that the dosing interval in multiple dosing schemes should be at least 2 weeks.

### 2.2. Toxicity in Dogs

**Figure 3:**
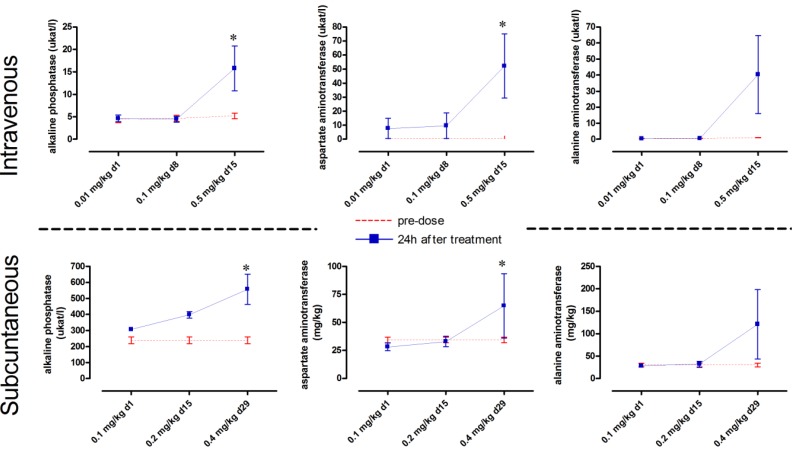
Monitoring of the liver enzymes alkaline phosphatase, aspartate aminotransferase and alanine aminotransferase) in dogs measured 24 h after (i) intravenous (IV) administration of weekly incremental doses of 0.01, 0.1 and 0.5 mg/kg (n = 4) and (ii) subcutaneous (SC) administration of weekly incremental doses of 0.1, 0.2 and 0.4 mg/kg (n = 4) * *p* < 0.05 (Two-way ANOVA with Bonferroni *post-hoc*)*.*

Intravenous dosing of maesabalide-III (study T4) and maesabalide IV (study T6) at 0.01 and 0.1 mg/kg did not result in overt adverse effects. Upon further dose-escalation to 0.5 mg/kg, the liver enzymes ALP, AST and ALT became increased within 24 h of dosing (Figure 3) and remained elevated after 2 weeks (data not shown). However, one male and one female had to be killed *in extremis* 7 days after dosing (day 22) because of significant body weight loss compared to pre-test BW (male: 8.6 to 7.3 kg; female: 6.6 to 5.5 kg) (Table S2), pale gingival mucosa and hypothermia. Gross pathology revealed pale discolouration of liver and kidneys and an enlarged spleen. Histopathological changes included moderate multifocal hepatocellular necrosis and vacuolization in scattered inflammatory foci. In the kidneys, tubular basophilia and minimal necrosis were present (Table S3). Almost identical results were obtained after IV dosing with maesabalide-IV, except that no animals needed to be killed (data not shown). Subcutaneous dosing of maesabalide-III (study T5) at 0.1 and 0.2 mg/kg induced no adverse effects. At 0.4 mg/kg, however, marginal toxicity was noted in terms of increased WBC-count and increased liver enzymes (Figure 3). These values slowly reversed to normal baseline values over the next 4 to 6 weeks (data not shown). The animals also developed a mild swelling at the injection site which appeared to be painful. Pathological examination revealed moderate to severe oedema and inflammation at the injection site.

These dog studies suggested that maesabalide-IV may be marginally less toxic than maesabalide-III as it did not seriously affect the clinical behaviour and growth of the dogs at comparable dose levels. The observed non-toxic intravenous dose level for both maesabalides was 0.01 mg/kg and only negligible effects were noted at 0.1 mg/kg. The liver is the prime target for toxicity with focal hepatocellular necrosis as the most prominent pathological effect. This is also reflected in the blood as a moderate increase of the liver enzymes within 24 h after dosing and persisting in a dose-related manner for up to 1 to 2 weeks. Several other plants containing saponins have been shown to be hepatotoxic. For example, increased ALT, AST and/or ALP levels were noted with extracts from *Anacardium occidentale* in rats [26], *Sida rhombifolia* and *Phytolacca dioica* in rats [27] and *Calycopteris floribunda* in calf, rabbit and rat [28]. Also the main metabolite of ginseng, compound K, induced a dose-dependent but reversible hepatotoxicity in dogs [29] similar to the maesabalide saponins in the present study.

Compared to the studies with PX-6518 in the mouse, it appears that the dog is far more susceptible to toxicity, as could partly be expected since large animal species are generally more sensitive to toxicity compared to small animals [30]. The enhanced toxicity may also be related to the fact that the dose-escalation was performed at weekly intervals, thereby inducing accumulation and hence toxicity of the drug.

### 2.3. Toxicokinetics in Dogs

**Figure 4:**
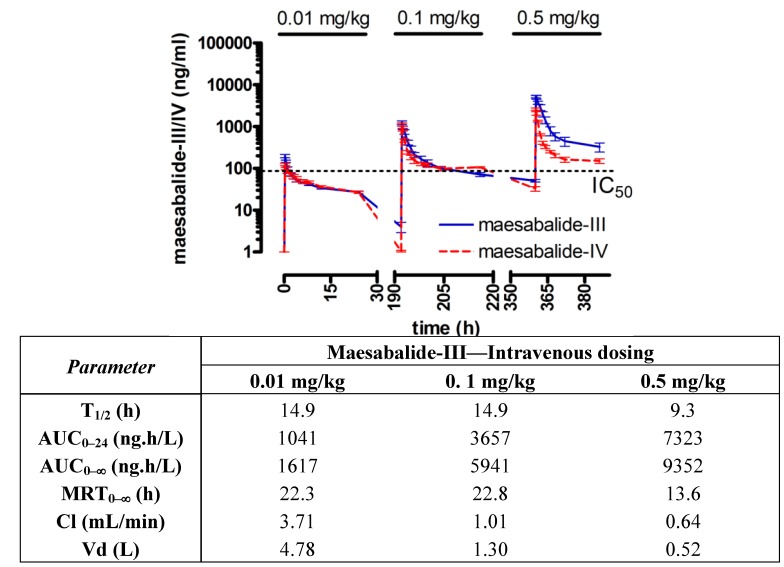
Plasma kinetics of maesabalide-III (n = 4) and maesabalide-IV (n = 4) after intravenous dose-escalation (0.01, 0.1 and 0.5 mg/kg at weekly intervals) in dogs. The dashed line represents the *in vitro* IC_50_ value of PX-6518 against intracellular amastigotes *Leishmania* *infantum* (taken from ref. [15]). Pharmacokinetic parameters were similar for both maesabalides and are listed for maesabalide-III in the table.

**Figure 5:**
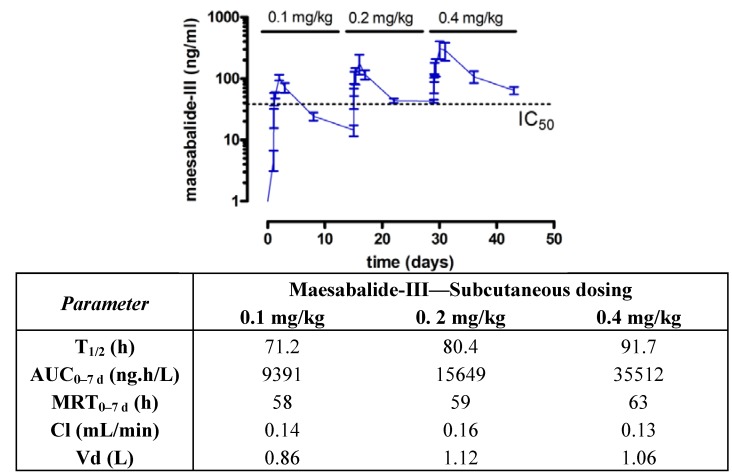
Plasma kinetics of maesabalide-III (n = 4) after subcutaneous dose-escalation (0.1, 0.2 and 0.4 mg/kg at weekly intervals) in dogs. The dashed line represents the *in vitro* IC_50_ value of PX-6518 against intracellular amastigotes *Leishmania* *infantum* (taken from ref [20]). Pharmacokinetic parameters are listed in the table.

Plasma kinetics of maesabalide-III and -IV after dose-escalating intravenous administrations revealed a non dose-linear systemic availability (AUC_0–24 h_) and elimination half-life of about 15 h. The overall clearance rate was very low, being 3.7 mL/min after 0.01 mg/kg and only 0.64 mL/min after 0.25 mg/kg (Figure 4). Subcutaneous dosing also led to accumulation of the compound, even after dosing at biweekly intervals (Figure 5). This is clearly illustrated by the fact that the pre-dose levels did not drop back to zero values. In fact, after a single dose at 0.1 mg/kg, plasma levels rapidly exceeded the minimal effective plasma concentration (IC_50_ = 40 ng/mL against *L. infantum*) and did not drop below this level until approximately 2 weeks after treatment (mean plasma level 24.3 ng/mL after 1 week and 14.4 after 2 weeks). Comparison of the elimination half-lives after intravenous (T_1/2_ 15 h) and subcutaneous dosing (T_1/2_ 71 h) suggests that the absorption from the injection site into the systemic circulation is rather slow and is limiting the overall elimination process. The moderate to severe oedema and inflammation may also have influenced the absorption process [31].

## 3. Experimental

### 3.1. PX-6518 Formulations

The extraction/purification of the active saponins consisted of two major steps: a solvent extraction of the biomass (dried and milled leaves of *M. balansae*), leading to “semi-purified” PX-6518, followed by a preparative chromatography leading to purified PX-6518, which actually consisted of a mixture of six saponins called maesabalides I to VI [14]. The exploratory toxicity studies were performed with purified PX-6518 in the mouse. Advanced toxicity evaluation was performed in beagle dogs using maesabalide-III and -IV, which had been shown to have the highest *in vitro* and *in vivo* antileishmanial activity [15].

### 3.2. Animals

The experiments were conducted in male (M) and female (F) SPF inbred CD-1 Swiss mice (Iffa Credo, Brussels, Belgium), 6 weeks old and weighing about 25 g. The pivotal toxicity studies with the individual maesabalides were performed in male and female beagle dogs of about 5–6 months old (BW: 8.3 kg for males and 7.3 kg for females). The studies in the dogs were carried out at Notox (Hertogenbosch, The Netherlands) and were kept under standard husbandry conditions. All interventions (dosing, blood sampling) were performed on fully conscious animals. All animal studies were cleared by the ethical committee at Tibotec (Mechelen, Belgium) that initiated the PX-6518 drug development programme. The studies were based on the ICH guideline “Non-Clinical Safety Studies for the Conduct of Human Clinical Trials for Pharmaceuticals” 16 July 1997, and the Note for Guidance concerning the application of Chapter I (B) of Part 2 of the Annex to Directive 75/318/EEC.

### 3.3. Study Design (Table 1)

#### 3.3.1. High-Dose Wash-Out Study in Mice (study T1)

A small-scale pilot trial was conducted to investigate the reversibility of toxicity parameters during a drug wash-out period after a 5-day loading session of PX-6518 at 20 mg/kg SC. PX-6518 was dissolved in isotonic 5% glucose vehicle at 5 mg/mL and filter-sterilized before use. Inbred mice (n = 42) were randomly divided into two groups. G1: vehicle control for 5 days, isotonic glucose (11M + 10F), G2: PX-6518 at 20 mg/kg BW for 5 days (11M + 10F). The formulation was dosed SC at 0.1 mL/25 g body weight at experiment days 0 to 4. Groups of 3 animals from each experimental group were killed for blood collection and determination of haematological and biochemical parameters on experiment days 7, 10, 14, 17, 21, 24 and 27. Blood was collected by axillary bleeding in heparin-coated tubes and immediately processed for haematological analysis. The plasma samples were stored at −20 °C until biochemical analysis. Pathological evaluation included assessing local irritation and gross pathology on spleen and liver for assessing toxic effects on the target organs.

#### 3.3.2. Low-Dose Wash-Out in Mice (Study T2, T3)

To evaluate the effects after single dosing at a lower tolerated dose level, a low-dose wash-out study was performed using 240 inbred mice that were randomly divided into 40 groups of 3 males and 3 females each. Groups of animals were killed for collection of blood at each evaluation time point at 7, 14, 21, 28 and 35 days. PX-6518 was dissolved in isotonic 5% glucose vehicle at 5 mg/mL, filter-sterilized before injection and dosed at 0.1 mL/25 g BW. The volume for injection was kept constant in the different dosing groups by dilution of the formulation in blank vehicle. A single SC or IV administration was performed on day-0 at 0, 1, 2.5 and 5 mg/kg BW. Blood was collected at each evaluation time for determination of haematological and serum biochemical parameters, as described for the high-dose wash-out study.

#### 3.3.3. Repeated-Dose Toxicity in Dogs

The studies were performed with maesabalide-III and maesabalide IV in male and female Beagle dogs. (Table S1). The non-toxic dose level in the mouse of 1 mg/kg BW was taken into consideration for dose-selection. Two types of dose-escalation studies were performed (i) IV dosing at weekly intervals with maesabalide-III and -IV and (ii) SC dosing at bi-weekly intervals with maesabalide-III only. Dogs (2M + 2F) were treated IV with escalating doses of 0.01, 0.1 and 0.5 mg/kg BW maesabalide-III (study T4) or maesabalide-IV (study T6). Full pathological examination was performed only on the killed animals while the toxicokinetic monitoring was done for maesabalide-IV only (re: complete set of animals, no deaths). The toxicity of maesabalide-III was also assessed after SC dosing (study T5). The volume for injection was kept constant at 0.3 mL/kg BW by appropriately diluting the stock formulation in blank vehicle. The successive SC injections were given every two weeks in the thoracic area to be able to check for local tolerance (swelling, redness, pain). The increase of liver enzymes was the primary parameter for evaluation of the systemic toxicity. 

### 3.4. Endpoint Measurements

Endpoints included clinical observations, post-mortem gross pathology, standard haematological analysis using microscopic counting (neutrophil count (NEU) and lymphocyte count (LYC)), spectrophotometer Cary 50 (Varian, Palo-Alto, CA, USA) (haemoglobin (Hb)) or the haematology Analyzers K-1000 (Sysmex, Hoeilaart, Belgium) (red blood cell count (RBC), white blood cell count (WBC), thrombocyte count (THR), haematocrit (HCT), mean corpuscular volume (MCV), mean corpuscular haemoglobin (MCH) and mean corpuscular haemoglobin concentration (MCHC)) and standard serum biochemical analysis using the Elan analyzer (Eppendorf, Rotselaar, Belgium) (total protein (TOP), albumin (ALB), glucose (GLU), cholesterol (CHO), triglycerides (TGL), blood urea nitrogen (BUN), alkaline phosphatase (ALP), aspartate aminotransferase (AST), alanine aminotransferase (ALT). 

### 3.5. Plasma Toxicokinetics

In the dog studies, plasma levels of the maesabalide-III and -IV were assessed using liquid chromatography combined with tandem mass spectrometry [14,32]. The analytes were extracted from plasma using acetonitrile (ACN). The deproteinized plasma was centrifuged and the clear supernatant was dried under a stream of nitrogen. The residue was resolved in methanol (MeOH), *bi*-distilled water and an internal standard (maesabalide-VI) was added. The resolved residue and the internal standard were trapped on a preconditioned C_18_ solid phase extraction cartridge and washed with MeOH/water mixture. After washing, the analyte and the internal standard were eluted from the cartridge using pure MeOH, then dried under a stream of nitrogen and resolved in injection solvent (MeOH:water 40:60 v/v). Chromatographic separation was carried out on an ODS-20 prodigy column using a 2690 high-performance liquid chromatograph from Waters. An injection volume (75 μL) of sample was kept in an autosampler set at 4 °C. The column temperature was maintained at 40 °C. The column was eluted at 0.6 mL/min with a mobile phase comprised of 90% 10 mM ammonium acetate in water/CAN/MeOH (1:1:3 v/v/v) and 10% 10 mM ammonium acetate in water with a runtime of 5 min. The mass spectrometer (PE Sciex API 3000) was operated under the positive ionization mode in electrospray MS/MS conditions (source temperature, 300 °C; ion source voltage 5,200 V). The flows of nebulizer, curtain and collision gases (nitrogen) were optimized to maximize the signal intensity S/N ratio), and data were acquired under multiple reaction monitoring incorporating a molecular weight scan from *m/z* 1,529.4 to *m/z* 683.4 for the maesabalide-III and -IV and from *m/z* 1,580.9 to *m/z* 339.1 for the internal standard. The concentrations of the maesabalides were calculated from the ratio of the peak areas of the component and the internal standard.

### 3.6. Statistical Analysis

Pre- and post-treatment data were statistically analysed using two-way ANOVA with Bonferroni *post-hoc*.

## 4. Conclusions

The present study in healthy mice and dogs demonstrates that high exposure of the antileishmanial triterpene saponin PX-6518 elicited marked toxic effects, such as increased neutrophils and liver enzymes upon repeated SC and IV administration. The pharmacokinetic data may suggest that therapeutically effective plasma levels could in fact be obtained at much lower dose levels that are devoid of any overt toxic side effects. However, this extrapolation between *in vitro* IC_50_ and observed plasma concentration must be interpreted with caution as this pharmacodynamic relation has not been established, in addition to the fact that the pharmacokinetics in *Leishmania*-infected subjects may also be altered in view of the disease-associated liver impairment.

From a practical treatment point of view, subcutaneous administration would be the sole approach, but local tolerance was rather poor, reflected by moderate to severe oedematous swelling and painful inflammation at the injection site. The latter could theoretically be tackled by using an adapted formulation rather than the simple aqueous vehicle that was used in this study. A much more relevant and pivotal problem was the occurrence of hepatotoxicity, for which the dog proved to be much more sensitive compared to the mouse. Increase of the liver enzymes AST, ALT, ALP and pathological changes such as hepatocellular necrosis and vacuolisation were the most prominent toxic signs. Despite apparent reversibility of these toxic effects, it was decided to stop the drug development programme of PX-6518 not only because of the nature of liver toxicity (hepatocellular necrosis), but also because of difficulties in manufacturing consistent batches of drug substance (unpublished information) and the fact that no projection can be given on how patients with visceral leishmaniasis would react to treatment with PX-6518, since the disease already impairs the liver function [33].

In summary, molecules with an established and highly promising proof-of-concept on activity in the laboratory still encounter many hurdles which preclude initiation of drug development programs. For neglected diseases, as is the case for leishmaniasis, this hurdle may even be higher in the absence of any prospect for ‘return of investment’. Fortunately, the public sector has now adequately taken over the initiative from the private sector [34,35].

## Supplementary Materials

Supplementary materials can be accessed at:  http://www.mdpi.com/1420-3049/18/4/4803/s1.

## References

[1] Bouarab K., Melton R., Peart J., Baulcombe D., Osbourn A. (2002). A saponin-detoxifying enzyme mediates suppression of plant defences. Nature.

[2] Osbourn A., Goss R.J., Field R.A. (2011). The saponins: Polar isoprenoids with important and diverse biological activities. Nat. Prod. Rep..

[3] Hill R.A., Connolly J.D. (2012). Triterpenoids. Nat. Prod. Rep..

[4] Hostettmann K., Marston A. (1995). Saponins. Chemistry & Pharmacology of Natural Products.

[5] Rao A.V., Gurfinkel D.M. (2000). The bioactivity of saponins: Triterpenoid and steroidal glycosides. Drug Metabol. Drug Interact..

[6] Price K.R., Johnson I.T., Fenwick G.R. (1987). The chemistry and biological significance of saponins in foods and feedingstuffs. Crit. Rev. Food Sci. Nutr..

[7] Stormer F.C., Reistad R., Alexander J. (1993). Glycyrrhizic acid in liquorice—Evaluation of health hazard. Food Chem Toxicol..

[8] Wee J.J., Mee Park K., Chung A.S., Benzie I.F.F., Wachtel-Galor S. (2011). Biological activities of ginseng and tts application to human health. Herbal Medicine: Biomolecular and Clinical Aspects.

[9] Attele A.S., Wu J.A., Yuan C.S. (1999). Ginseng pharmacology: Multiple constituents and multiple actions. Biochem. Pharmacol..

[10] Wang Z.Y., Nixon D.W. (2001). Licorice and cancer. Nutr. Cancer.

[11] Sirtori C.R. (2001). Aescin: Pharmacology, pharmacokinetics and therapeutic profile. Pharmacol. Res..

[12] Widgerow A.D., Chait L.A., Stals R., Stals P.J. (2000). New innovations in scar management. Aesthetic Plast. Sur..

[13] Cox J.C., Sjolander A., Barr I.G. (1998). ISCOMs and other saponin based adjuvants. Adv. Drug Deliv. Rev..

[14] Leonard S., Capote R., Germonprez N., van Puyvelde L., de Kimpe N., Vermeersch M., Rosier J., Maes L., Roets E., Hoogmartens J. (2003). Liquid chromatographic method for analysis of saponins in *Maesa balansae* extract active against leishmaniasis. J. Chromatogr. A.

[15] Maes L., Vanden Berghe D., Germonprez N., Quirijnen L., Cos P., de Kimpe N., van Puyvelde L. (2004). *In vitro* and *in vivo* activities of a triterpenoid saponin extract (PX-6518) from the plant *Maesa balansae* against visceral *Leishmania* species. Antimicrob. Agents Chemother..

[16] Germonprez N., Maes L., van Puyvelde L., van Tri M., Tuan D.A., de Kimpe N. (2005). *In vitro* and *in vivo* anti-leishmanial activity of triterpenoid saponins isolated from *Maesa balansae* and some chemical derivatives. J. Med. Chem..

[17] Vermeersch M., Foubert K., Inocencio da Luz R.A., van Puyvelde L., Pieters L., Cos P., Maes L. (2009). Selective antileishmania activity of 13,28-epoxy-oleanane and related triterpene saponins from the plant families Myrsinaceae, Primulaceae, Aceraceae and Icacinacea. Phytother. Res..

[18] Maes L., Germonprez N., Quirijnen L., van Puyvelde L., Cos P., Vanden Berghe D. (2004). Comparative activities of the triterpene saponin maesabalide III and liposomal amphotericin B (AmBisome) against *Leishmania donovani* in hamsters. Antimicrob. Agents Chemother..

[19] Inocencio da Luz R.A., Vermeersch M., Deschacht M., Hendrickx S., van Assche T., Cos P., Maes L. (2011). *In vitro* and *in vivo* prophylactic and curative activity of the triterpene saponin PX-6518 against cutaneous *Leishmania* species. J. Antimicrob. Chemother..

[20] Apers S., Baronikova S., Sindambiwe J.B., Witvrouw M., de Clercq E., Vanden Berghe D., van Marck E., Vlietinck A., Pieters L. (2001). Antiviral, haemolytic and molluscicidal activities of triterpenoid saponins from *Maesa lanceolata*: Establishment of structure-activity relationships. Planta Med..

[21] Gao S., Basu S., Yang Z., Deb A., Hu M. (2012). Bioavailability challenges associated with development of saponins as therapeutic and chemopreventive agents. Curr. Drug Targets.

[22] Yu K., Chen F., Li C. (2012). Absorption, disposition, and pharmacokinetics of saponins from Chinese medicinal herbs: What do we know and what do we need to know more?. Curr. Drug Metab..

[23] Podolak I., Galanty A., Sobolewska D. (2010). Saponins as cytotoxic agents: A review. Phytochem. Rev..

[24] Cos P., Vlietinck A., Vanden Berghe D., Maes L. (2006). Demonstration of anti-infective potential in natural products: How to develop a stronger *in vitro* “proof of concept”. J. Ethnopharmacol..

[25] Böttger S., Hofmann K., Melzig M.F. (2012). Saponins can perturb biologic membranes and reduce the surface tension of aqueous solutions: A correlation?. Bioorg. Med. Chem..

[26] Okonkwo T.J., Okorie O., Okonta J.M., Okonkwo C.J. (2010). Sub-chronic hepatotoxicity of *Anacardium occidentale* (Anacardiaceae) inner stem bark extract in Rats. Indian J. Pharm. Sci..

[27] Ashafa A.O., Sunmonu T.O., Afolayan A.J. (2010). Toxicological evaluation of aqueous leaf and berry extracts of *Phytolacca dioica* L. in male Wistar rats. Food Chem. Toxicol..

[28] Sreekanth P., Narayana K., Shridhar N.B., Bhat A. (2006). Toxicity studies of *Calycopteris floribunda Lam.* in calf, rabbit and rat. J. Ethnopharmacol..

[29] Gao Y.L., Liu Z.F., Li C.M., Shen J.Y., Yin H.X., Li G.S. (2011). Subchronic toxicity studies with ginsenoside compound K delivered to dogs via intravenous administration. Food Chem. Toxicol..

[30] Goldsmith M.A., Slavik M., Carter S.K. (1975). Quantitative prediction of drug toxicity in humans from toxicology in small and large animals. Cancer Res..

[31] McDonald T.A., Zepeda M.L., Tomlinson M.J., Bee W.H., Ivens I.A. (2010). Subcutaneous administration of biotherapeutics: Current experience in animal models. Curr. Opin. Mol. Ther..

[32] Foubert K., Vermeersch M., Theunis M., Apers S., Cos P., Claeys M., van Puyvelde L., Pieters L., Maes L. (2009). LC/MS analysis of 13,28-epoxy-oleanane saponins in *Maesa* spp. extracts with antileishmanial activity. Phytochem. Anal..

[33] Mathur P., Samantaray J.C., Samanta P. (2008). High prevalence of functional liver derangement in visceral leishmaniasis at an Indian tertiary care center. Clin. Gastroenterol. Hepatol..

[34] Croft S.L. (2005). Public-private partnership: From there to here. Trans. R. Soc. Trop. Med. Hyg..

[35] Ioset J.R., Chang S. (2011). Drugs for Neglected Diseases initiative model of drug development for neglected diseases: Current status and future challenges. Future Med. Chem..

