# Supplementary material for: Intravenous and Subcutaneous Toxicity and Absorption Kinetics in Mice and Dogs of the Antileishmanial Triterpene Saponin PX-6518
Source: Molecules. 2013 Apr 22;18(4):4803–15. doi: 10.3390/molecules18044803 (PMC6269738; doi:10.3390/molecules18044803)
Supplement: Supplementary file 1 [file molecules-18-04803-s001.pdf]

# Supplementary Materials

**Table S1.** Standard experimental protocol in the range finding dog toxicity studies.

| **Test system** | | |
|---|---|---|
| Animal species | Beagle dog, pure bred | |
| Source | Marschall Farms, New York, NY, USA | |
| Age at start | approx 5–6 months | |
| **Husbandry** | | |
| Conditions | room temp, RH 40–80%, 12 h night-day cycle | |
| Accomodation | individual in stainless steel cages | |
| Diet | standard dog maintenance pelleted food (Altromin diet 4029) | |
| Water | tap water *ad libitum* | |
| **Study design** | **Intravenous studies (T4 and T6)** | **Subcutaneous study (T5)** |
| Number of animals | 2 males, 2 females | 2 males, 2 females |
| Treatment days | day 1 (0.01 mg/kg); day 8 (0.1 mg/kg); day 15 (0.5 mg/kg) | day 1 (0.1 mg/kg); day 15 (0.2 mg/kg); day 29 (0.4 mg/kg) |
| Clinical signs | daily | daily |
| Body weights | days 1, 8, 15, 22 | days 1, 8, 15, 22, 29, 36 |
| Food/water consumption | daily | daily |
| Haematology & biochemistry | days 1, 8, 15, 22 | days 2, 8, 15, 22, 29, 36 |
| Urinalysis | days 1, 8, 15 | days 1, 15, 29 |
| Toxicokinetics | days 1, 8, 15 | days 1, 15, 29 |
| Necropsy | only in 2 animals that were killed in study T4 | not done |

**Table S2.** Evolution of body weight (kg) after intravenous dosing (studies T4, T6).

| **Dog** | **Study T4** | | | | | **Study T6** | | | | |
|---|---|---|---|---|---|---|---|---|---|---|
| | **pre-test** | **1** | **8** | **15** | **22** | **pre-test** | **1** | **8** | **15** | **22** |
| **M1** | 8.60 | 9.0 | 9.2 | 8.8 | 7.3# | 8.07 | 8.08 | 7.80 | 7.98 | 7.63 |
| **M2** | 7.85 | 7.9 | 9.7 | 7.8 | 7.6 | 8.45 | 8.75 | 8.62 | 8.84 | 8.21 |
| *average* | *8.23* | *8.45* | *9.47* | *8.33* | *7.42* | *8.26* | *8.42* | *8.21* | *8.41* | *7.92* |
| *sd* | *0.53* | *0.74* | *0.35* | *0.71* | *0.19* | *0.27* | *0.47* | *0.58* | *0.61* | *0.41* |
| **F1** | 6.6 | 6.5 | 6.5 | 6.5 | 5.2# | 7.52 | 7.92 | 7.99 | 8.21 | 8.22 |
| **F2** | 8.0 | 8.2 | 8.4 | 8.0 | 7.6 | 7.03 | 7.34 | 7.20 | 7.29 | 7.07 |
| *average* | *7.28* | *7.37* | *7.46* | *7.27* | *6.38* | *7.28* | *7.63* | *7.60* | *7.75* | *7.65* |
| *sd* | *1.02* | *1.23* | *1.36* | *1.09* | *1.69* | *0.35* | *0.41* | *0.56* | *0.65* | *0.81* |

# M1 and F1 needed to be killed in extremis due to serious side effects.

**Table S3.** Major clinical and pathological findings (study T4).

| |
|---|
| *Clinical signs and mortality*<br>One male (M1) and one female (F1) were killed in extremis 7 days after treatment with 0.5 mg/kg due to severe toxic side effects. Clinical signs included calm behavior, decreased locomotor activity, a dull fur, vomiting of mucus, diarrhoea, salivation, purulent discharge from the eyes and an emaciated appearance at 4–7 days after drug administration. This was also reflected in both the absolute and relative food consumption (*i.e.*, after correction for body weight). No effect was noted on water consumption.<br><br>*Pathology*<br>Yellow to pale discoloration was observed for the liver, kidney, salivary glands and body fat. Both the liver and the kidneys exhibited clear morphological alterations indicative of maesabalide toxicity. The liver alterations were characterized by multifocal hepatocellular necrosis (moderate severity), vacuolization (slight to moderate) and inflammatory cell foci (moderate), cholestasis (moderate) and intra-hepatocellular basophilic aggregates (slight). The kidney findings included tubular basophilia (moderate to severe), tubular necrosis (slight) and mineralized granular casts. |
